# CD40 Agonism in Pancreatic Ductal Adenocarcinoma: Expression, Biology, and Therapeutic Targeting

**DOI:** 10.3390/cancers18111743

**Published:** 2026-05-27

**Authors:** Songul Kucukcelebi, Aniek E. van Diepen, Judith de Vos-Geelen, Casper H. J. van Eijck, Nadine van Montfoort, Casper W. F. van Eijck

**Affiliations:** 1 Solid Tumor Immunology Research Rotterdam (STIRR) Group, Department of Pulmonary Medicine, Erasmus University Medical Center, 3015 GD Rotterdam, The Netherlands; 2 Department of Internal Medicine, Division of Medical Oncology, GROW Research Institute for Oncology & Reproduction, Maastricht University Medical Center+, 6202 AZ Maastricht, The Netherlands; 3 Department of Gastroenterology and Hepatology, Leiden University Medical Center, 2300 RC Leiden, The Netherlands

**Keywords:** pancreatic cancer (PDAC), CD40-agonists, tumor microenvironment, immunotherapy

## Abstract

Pancreatic ductal adenocarcinoma (PDAC) remains highly lethal and largely refractory to immune checkpoint inhibition because limited antigen-specific priming, myeloid suppression, dense desmoplasia, and abnormal vasculature enforce immune exclusion. CD40 links CD4+ T-cell help through CD40L/CD154 to antigen-presenting-cell (APC) licensing and CD8+ T-cell priming, making CD40 agonism a rational strategy to stimulate antitumor immunity in PDAC. CD40 is expressed on APCs and has also been reported on subsets of PDAC tumor cells, cancer-associated fibroblasts, and endothelial cells, indicating that CD40 agonists may affect immune activation, stromal/vascular remodeling, and context-dependent tumor-cell-intrinsic signaling. TRAF-dependent CD40 signaling activates canonical and non-canonical NF-kB, MAPK, and PI3K/AKT pathways, promoting APC maturation, IL-12-associated Th1 programming, macrophage repolarization, and matrix remodeling; tumor-intrinsic effects remain more variable, ranging from apoptotic to pro-survival programs. Clinically, CD40 agonists have shown pharmacodynamic immune engagement and manageable toxicity, mainly in combinations with chemotherapy, checkpoint inhibitors, and vaccine platforms, but efficacy remains inconsistent, and randomized validation is incomplete. Baseline CD40 expression has not reliably predicted benefit. Future development should prioritize spatially resolved tumor-immune profiling, longitudinal pharmacodynamic biomarkers, optimized sequencing, and agent-specific dosing strategies. This review integrates CD40 expression, signaling, and clinical evidence in PDAC to support more rational, biomarker-guided development of CD40-directed immunotherapy.

## Simple Summary

Pancreatic ductal adenocarcinoma (PDAC) is difficult to treat and usually does not respond to current immunotherapies. A major barrier is its dense, myeloid-rich, and suppressive tumor microenvironment, which limits immune cell infiltration and effective tumor killing. CD40 is an immune receptor that can activate antigen-presenting cells, support T-cell priming, and remodel myeloid and stromal compartments. This review summarizes CD40 expression in PDAC, the biology of CD40 signaling, and the clinical development of CD40 agonists. It also explains why combinations with chemotherapy, checkpoint blockade, or cancer vaccines are probably required, and why baseline CD40 expression alone is unlikely to select patients. Better spatial and pharmacodynamic biomarkers should support more rational trials without overstating current clinical benefit.

## 1. Introduction

Pancreatic ductal adenocarcinoma (PDAC), the predominant form of pancreatic cancer, accounts for 4.8% of global cancer deaths [1]. In 2022, pancreatic cancer caused approximately 467,000 deaths and was the sixth leading cause of cancer mortality worldwide, despite ranking twelfth by incidence with approximately 511,000 new cases [1]. Incidence is increasing, with the highest age-standardized rates reported in Europe, North America, and Australia, and projections suggest that pancreatic cancer may become the second leading cause of cancer-related death by 2030 [2]. Standard combination chemotherapy, including FOLFIRINOX and gemcitabine/nab-paclitaxel, provides limited long-term benefit [3,4]; median survival remains poor, and 5-year survival is approximately 13% [5]. New treatment strategies are therefore urgently needed.

Immune checkpoint inhibitors (ICIs) have transformed treatment in several solid tumors [6,7,8,9,10,11,12,13,14], but they have limited activity in biomarker-unselected PDAC [15,16,17]. Resistance reflects a suppressive tumor microenvironment (TME) enriched for tumor-associated macrophages, myeloid-derived suppressor cells, and regulatory T cells, along with dense desmoplasia and poorly perfused vasculature, which restrict immune trafficking and drug delivery. Low tumor mutational burden, sparse tumor-specific effector T cells, and paucity of dendritic cells (DCs) further reinforce an immunologically cold phenotype [18,19,20,21]. Effective immunotherapy in PDAC will therefore likely require both improved antigen presentation and T-cell priming, as well as relief of myeloid-stromal constraints on effector function.

CD40, a tumor necrosis factor receptor (TNFR) superfamily member, is a central co-stimulatory receptor expressed mainly on antigen-presenting cells (APCs), including DCs, macrophages, and B cells [22,23,24]. Ligation by CD40L (CD154), primarily expressed on activated CD4+ T cells, induces APC maturation and licenses DCs to cross-present tumor antigens to T cells [22,23,24,25,26,27]. CD40L can also be supplied in soluble form, including by platelets, linking the pathway to immune and vascular biology [22,23].

CD40 has also been reported on subsets of PDAC tumor cells, with heterogeneous expression across tumors and disease stages [28,29], and in stromal compartments, including cancer-associated fibroblasts (CAFs) and selected endothelial populations [27,29,30,31,32]. Thus, CD40-directed therapy may act beyond classical APC activation and affect tumor-intrinsic, stromal, and vascular programs.

Several CD40 agonists are in clinical development and have shown pharmacodynamic immune activation with generally acceptable safety across solid tumors, including PDAC [33,34,35]. However, CD40 signaling is context-dependent: APC activation is typically immunostimulatory, whereas tumor-intrinsic signaling may support either antitumor or pro-survival programs depending on cellular state and microenvironmental cues [36,37,38]. This complexity has direct implications for therapeutic design and patient selection.

Here, we review CD40 expression, measurement considerations, signaling biology, and cell-specific functions in PDAC, and summarize the clinical development of CD40-directed strategies. We focus on how combinations, sequencing, and biomarkers may help convert pharmacodynamic immune activation into reproducible clinical benefit.

## 2. CD40 Expression Landscape in PDAC

In PDAC, CD40 is most consistently detected on immune cells, but its expression has also been reported in the tumor and stromal compartments. This multicompartment distribution is relevant because CD40 agonism may simultaneously influence immune activation, stromal remodeling, vascular inflammation, and tumor-cell biology.

### 2.1. CD40 Expression on Non-Immune PDAC Compartments

Beyond immune cells, CD40 is detectable in PDAC tumor cells, CAFs, and endothelial cells. This distribution supports a broader therapeutic model in which CD40 agonists not only license APCs but may also influence tumor-intrinsic and stromal/vascular programs.

A substantial subset of PDAC tumor cells expresses CD40, with reported positivity of approximately 68% across cohorts [28,29]. Expression is heterogeneous within tumors and may be higher in advanced or metastatic disease [28]. Tumor-intrinsic CD40 signaling is context-dependent and can drive either apoptosis or proliferation/survival, with NF-kappaB and MAPK as key downstream effectors [36,37,38]. Tumor-cell CD40 has also been linked to immune evasion by reducing T-cell CD154 (CD40L) expression, suppressing cytokine production, and decreasing T-cell proliferation [39]. These divergent effects likely reflect oncogenic state, cytokine context, and spatial differences between primary tumors and metastases [40,41]. Thus, tumor-cell CD40 may influence both direct tumor signaling and immunomodulatory efficacy, but baseline expression alone should not be treated as a reliable response biomarker in PDAC.

Within the stromal compartment, CD40 expression on subsets of CAFs and endothelial cells links the pathway to extracellular matrix (ECM) remodeling and vascular regulation. CD40 activation on CAFs can induce cytokine and growth-factor programs that influence matrix metalloproteinase (MMP) activity, tumor invasion, and ECM remodeling in a subtype- and context-dependent manner [32,42]. These stromal effects are particularly relevant in PDAC, where dense desmoplasia forms a major physical and immunological barrier to immune-cell infiltration and drug delivery.

CD40-expressing endothelial cells participate in inflammatory vascular remodeling and angiogenic processes [22,23,38,43,44]. CAF–endothelial crosstalk through factors such as vascular endothelial growth factor (VEGF) and hepatocyte growth factor (HGF) contributes to hypovascularity, impaired drug delivery, and immune exclusion in PDAC [45,46,47]. CD40 signaling may therefore influence the accessibility of the TME to immune cells and therapeutic agents.

Consistent with these compartmental effects, agonistic CD40 activation can induce stromalysis via proteases produced by activated myeloid cells, which degrade dense ECM and facilitate DC and effector T-cell infiltration [30,31,42]. This stromal effect is a key rationale for CD40 agonists in PDAC, as it addresses a major barrier to immune activation and drug delivery. Overall, CD40-directed therapies may act through APC activation, myeloid reprogramming, and modulation of tumor-intrinsic and stromal/vascular programs.

### 2.2. CD40 Expression on Immune Cells Within the PDAC Tumor Microenvironment

The strongest and most reproducible CD40 expression in PDAC is found on APC populations, including DCs, macrophages, monocytes, and B cells [22,23,24]. This immune-cell expression provides the core biological rationale for therapeutic CD40 agonism in a disease characterized by profound myeloid suppression and weak endogenous priming.

DC subsets involved in cross-priming, including cDC1 and cDC2 populations, CD141+ DCs, and LAMP3+ mature/regulatory DC states, require CD40 engagement for functional maturation and efficient CD8+ T-cell priming [23,26,48]. In PDAC, DC function is often inhibited by tumor-derived cytokines and suppressive myeloid signals, resulting in inadequate T-cell priming [49]. CD40 agonists aim to bypass insufficient endogenous CD40L signaling and restore APC function.

Tumor-associated macrophages (TAMs), frequently skewed toward suppressive M2-like states, express variable CD40 levels and are important CD40 agonist targets [50]. CD40 activation can reprogram TAMs toward inflammatory, antigen-presenting phenotypes with increased IL-12 and TNF-alpha production and induction of matrix-degrading programs [42]. These macrophage effects link immune activation to stromal remodeling and may help overcome the ECM barrier that restricts immune infiltration and drug delivery in PDAC [30].

B cells in tertiary lymphoid structures (TLS) also express CD40 and may support local antigen presentation and T-cell activation. In mature TLS, CD40 signaling promotes antigen-driven B-cell maturation, plasma-cell differentiation, and local antibody production, thereby supporting adaptive antitumor immunity [51].

Activated CD4+ T cells are the main source of CD40L, which is transiently upregulated after T-cell activation. CD40L-CD40 engagement licenses APCs to enhance antigen presentation and coordinate innate and adaptive immune activation [22,25,27]. Because PDAC is characterized by dysfunctional T-cell responses and insufficient immune activation, CD40 agonists aim to bypass this limitation by directly activating CD40 signaling.

CD40 expression has also been reported on subsets of myeloid-derived suppressor cells (MDSCs), in which CD40 engagement may modulate suppressive or effector functions in a context-dependent manner [52,53,54].

NK cells express CD40L rather than the CD40 receptor. CD40L-dependent NK-cell activation can support the recognition and killing of CD40-expressing target cells through activation of the NF-kappaB pathway [55,56] and is regulated by IL-12 and IFN-gamma [57,58]. NK cells may therefore contribute to the elimination of CD40-expressing tumor cells and amplification of antitumor immunity.

Together, CD40 effects on DCs, macrophages, B cells, NK cells, and other immune populations provide a mechanistic basis for converting the suppressive PDAC TME into a more inflamed and therapy-responsive state.

## 3. Biological Functions and Signaling Pathways of CD40 in PDAC

**Figure 1:**
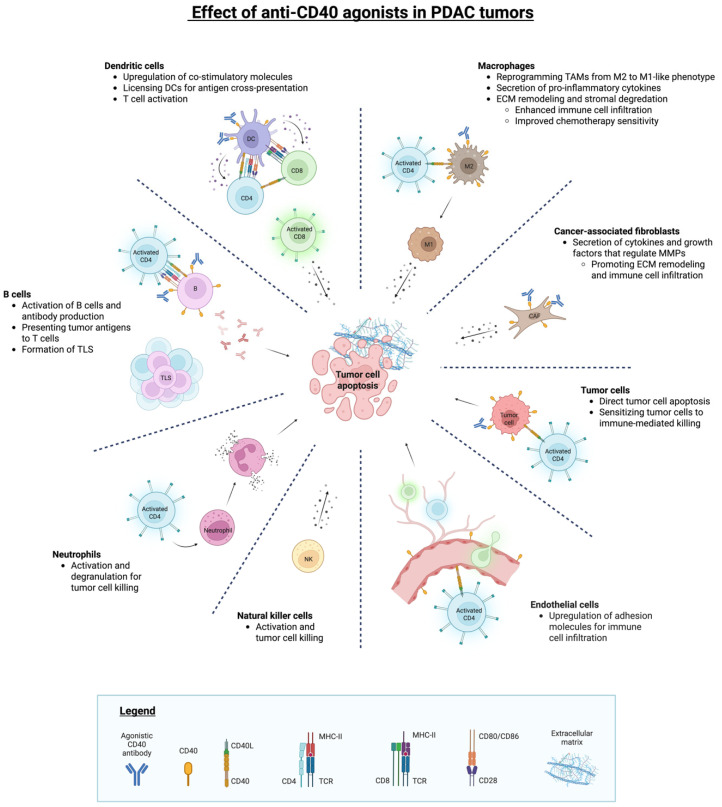
Expression of CD40 and effect of agonistic CD40 antibodies in pancreatic cancer.

CD40 biology in PDAC is compartment-dependent and shaped by the CD40-expressing cell type, local ligand availability, and inflammatory context. The key cellular and molecular mechanisms engaged by CD40 agonists are summarized in Figure 1.

### 3.1. Core CD40 Signaling Pathways Shaping Immune Activation

CD40 lacks intrinsic enzymatic activity and signals through recruitment of TNF receptor-associated factor (TRAF) adaptor proteins after ligand-induced receptor clustering. TRAF composition and stoichiometry determine downstream pathway bias and functional output [59,60]. Canonical NF-kB activation is a central consequence of CD40 ligation and induces transcriptional programs that support APC maturation and T-cell priming, including cytokines such as IL-6 and TNF-alpha, adhesion molecules such as ICAM-1, and B7-family co-stimulatory regulators [60,61].

CD40 engagement upregulates CD80 (B7-1) and CD86 (B7-2) on DCs and B cells, strengthening T-cell activation [25,26,62,63]. It can also increase B7-H3 (CD276) expression on DCs, and this upregulation was functionally required for enhanced antitumor immunity induced by CD40-activated, tumor antigen-pulsed DCs [60,64,65]. Because B7-H3 is overexpressed in several solid tumors, including PDAC, and is linked to progression, metastasis, and treatment resistance [66,67], it has become an immunotherapeutic target. Early-phase B7-H3 antibody–drug conjugates such as YL201 and HS-20093 (GSK5764227) are therefore relevant to this axis [68,69]. Whether CD40-induced B7-H3 upregulation enhances ADC targeting or instead represents compensatory immune escape remains unresolved.

CD40 also engages non-canonical NF-kB signaling, supporting more sustained APC and lymphoid-organization programs [70]. In parallel, CD40 activates MAPK cascades, including ERK, JNK, and p38, which regulate cytokine production, differentiation, and survival across myeloid and stromal cells [60,64,65], and PI3K/AKT signaling, which supports APC metabolism and antigen processing while modulating survival in a context-dependent manner [71]. JAK3-STAT5 is not generally considered a primary direct CD40 pathway but may be engaged indirectly through CD40-induced autocrine or paracrine cytokines, thereby tuning inflammatory polarization and APC tolerogenicity [72,73,74,75]. These pathways explain why CD40 agonism can produce different outputs across PDAC compartments.

The JAK/STAT pathway, particularly STAT3, is implicated in pancreatic cancer inflammation, progression, and immune regulation [76,77,78,79,80,81]. Clinical translation of JAK inhibition in PDAC has been disappointing. A randomized phase II trial suggested benefit from ruxolitinib plus capecitabine in a subgroup with systemic inflammation [82], but the subsequent phase III JANUS 1 and JANUS 2 trials in advanced/metastatic PDAC were stopped for futility [83]. Itacitinib, a selective JAK1 inhibitor, combined with gemcitabine and nab-paclitaxel, showed some activity in a phase Ib/II study, including an overall response rate of 24%, but development was also stopped after the negative JANUS results [84].

Because CD40 agonism may indirectly engage JAK3-STAT5 signaling [72,73,74,75], combined CD40 and JAK/STAT modulation is mechanistically plausible but clinically uncertain. In theory, CD40 activation could improve APC licensing and T-cell priming, while JAK/STAT inhibition could reduce tumor-promoting inflammatory signaling and chemotherapy resistance. In practice, JAK inhibitors can also suppress T-cell and DC function [85,86,87]. Therefore, whether JAK/STAT blockade would enhance or impair CD40 agonist efficacy requires dedicated preclinical testing before clinical development.

### 3.2. The CD40-CD40L Axis In Vivo: Ligand Sources and Signaling Context

Physiologic CD40 activation is mediated by CD40L (CD154), which is transiently expressed on activated CD4+ T cells and is required for APC licensing and cytotoxic T-cell priming [22,23,25,26]. CD40L can also be supplied by other activated immune cells and in soluble form, especially by platelets, linking CD40 signaling to vascular inflammation and endothelial activation [22,23,38,43,44]. The biological outcome depends on CD40 expression, the source and form of CD40L (cell-bound versus soluble), and the local inflammatory milieu [65,88]. In PDAC, where productive CD4+ help is often limited and myeloid suppression is dominant, therapeutic CD40 agonism aims to bypass inadequate endogenous CD40L and restore licensing signals [23,26,63].

### 3.3. CD40-Driven Activation of Antigen-Presenting Cells

CD40 engagement on DCs, macrophages, and B cells induces maturation and bridges innate and adaptive immunity. In DCs, CD40 ligation through CD40L or agonistic antibodies upregulates MHC class I/II and enhances cross-presentation, enabling CD4+ and CD8+ T-cell priming [23,26]. CD40 activation also increases CD80/CD86 expression [26,63] and IL-12 production, supporting Th1 polarization, CD8+ T-cell expansion, and IFN-gamma-associated effector programming [23,26,42,89]. Because cross-priming is profoundly deficient in untreated PDAC [21,90,91], restoration of this axis is a central goal of CD40 agonist therapy.

### 3.4. Reprogramming of Tumor-Associated Macrophages

The PDAC TME favors the emergence of suppressive TAM states driven by chronic inflammation, hypoxia, and stromal cues [50,92,93]. CD40 signaling can reprogram M2-like macrophages toward inflammatory, antigen-presenting phenotypes with increased IL-12/TNF-alpha production, improved antigen presentation, matrix-degrading programs, and enhanced immune-cell recruitment [30,31,42,94]. In preclinical PDAC models, macrophage reprogramming is a major determinant of CD40 agonist activity and is linked to improved T-cell infiltration and intratumoral activation [30,95,96].

### 3.5. Stromal and Vascular Remodeling Downstream of CD40 Activation

CD40 agonism can loosen PDAC stromal barriers through myeloid-driven remodeling [31]. CD40-activated macrophages infiltrate tumors, acquire tumoricidal functions, and deplete stromal components that block immune-cell entry [30]. These macrophages increase MMP expression and may suppress profibrotic CAF activity through paracrine and contact-dependent mechanisms, reducing ECM deposition and improving immune trafficking and drug penetration [30,31,42]. Endothelial CD40 is upregulated by inflammatory stimuli such as TNF-alpha and IFN-gamma [43,97]. CD40-CD40L signaling in endothelium triggers inflammatory adhesion and cytokine programs [43,44,98]. This includes adhesion molecules, inflammatory cytokines, chemokines, MMPs, and procoagulant activity [43,98,99]. In PDAC models, inflammatory remodeling is associated with increased DC and T-cell infiltration and may help overcome physical and cellular barriers to immunity [27,30,42,94,100]. The vascular consequences are complex: CD40-CD40L signaling can induce angiogenic factors such as VEGF and fibroblast growth factor [101,102,103], and studies have reported both VEGF-driven neoangiogenesis [104,105] and reduced tumor vasculature with slower growth after inflammatory remodeling [38,106].

### 3.6. Tumor-Intrinsic CD40 Signaling in PDAC

In subsets of PDAC, malignant epithelial cells express CD40 and can transmit tumor-intrinsic signals after ligation [28,29,107]. NF-kB and MAPK activation have been linked to divergent outcomes, including apoptosis [28,108] and enhanced proliferation or survival [107,109,110], depending on cellular state and microenvironmental context [110,111]. Tumor-cell CD40 signaling may also attenuate antitumor immunity by reducing T-cell CD154 expression and impairing cytokine production and proliferation [39,107,112]. The net effect of CD40 agonism may therefore reflect the balance between APC licensing and myeloid reprogramming, on the one hand, and tumor-cell signaling, on the other [24,32,113]. Future CD40 trials should account for tumor-cell CD40 expression and downstream signaling competence.

## 4. Therapeutic Targeting of CD40 in PDAC

CD40 agonism is pursued in PDAC to replace the insufficient endogenous CD40L-driven APC licensing and to activate programs deficient in this disease: antigen presentation, T-cell priming, myeloid reprogramming, and remodeling of stromal/vascular barriers. Because monotherapy has produced robust pharmacodynamic but variable clinical effects, development has shifted toward rational combinations and biomarkers that capture effective pathway engagement.

### 4.1. CD40 Agonistic Modalities and Design Principles

Productive CD40 signaling requires receptor clustering. In vivo agonism, therefore, depends on epitope, valency, and, for monoclonal antibodies, Fc-gamma receptor (Fc-gammaR)-mediated crosslinking on accessory cells [114]. Isotype, Fc engineering, Fc-gammaR engagement, and binding geometry shape immune activation, tissue distribution, tolerability, and dosing schedule. CD40 agonists should therefore not be treated as interchangeable. Agents evaluated in pancreatic cancer include selicrelumab (RG7876/CP-870,893), SEA-CD40, mitazalimab (JNJ-64457107/ADC-1013), sotigalimab (APX005M), CDX-1140, ChiLob 7/4, and LVGN7409 (Table 1). Across agents, monotherapy activity in PDAC has been modest, supporting combinations that increase antigen release or relieve downstream suppression.

### 4.2. Combination Approaches with Chemotherapy

Chemotherapy provides a mechanistic foundation for CD40 agonism by increasing antigen availability through tumor-cell death, promoting immunogenic cell-death signals, and altering myeloid composition. CD40-induced myeloid and stromal remodeling may also improve tumor permeability and drug access [31,95,115]. The central goal of chemo-CD40 therapy is to shift CD163+ and other suppressive macrophage populations toward inflammatory, antigen-presenting, and matrix-remodeling states. This is clinically relevant because high levels of CD163+ macrophages are associated with poor outcomes across cancers [90,116,117]. Preclinical models support synergy between chemotherapy and CD40 activation, with improved T-cell priming and survival [31,95,115].

Clinical chemotherapy–CD40 combinations in PDAC have used gemcitabine, gemcitabine/nab-paclitaxel, and mFOLFIRINOX backbones (Table 2). In chemotherapy-naive advanced PDAC, selicrelumab plus gemcitabine produced an ORR of 19% (4/22), a median PFS of 5.2 months, a median OS of 8.4 months, and a 1-year OS of 28.6%, with inflammatory cytokine induction and increased B-cell costimulatory molecules [118].

In resectable PDAC, neoadjuvant selicrelumab with or without gemcitabine/nab-paclitaxel, followed by adjuvant gemcitabine/nab-paclitaxel and selicrelumab, showed clear pharmacodynamic remodeling. In the selicrelumab-only neoadjuvant group (n = 11), median DFS and OS were 9.8 and 23.4 months, respectively. In the combination neoadjuvant group (n = 5), median DFS and OS were not reached; across both groups, median DFS was 13.9 months and median OS was 23.4 months. Treated tumors were more often T-cell-enriched than untreated tumors (82% vs. 37%, *p* = 0.004), with more active/proliferative T cells, reduced fibrosis, fewer M2-like macrophages, and increased mature DCs [42].

In PRINCE, sotigalimab (APX005M) was combined with gemcitabine/nab-paclitaxel in untreated metastatic PDAC. The phase Ib portion showed responses in 14/24 DLT-evaluable patients (58%) [122]. In the randomized phase II portion (n = 105), the primary endpoint of 1-year OS was met for nivolumab/chemotherapy (57.7%, *p* = 0.006 versus a 35% historical rate), but not for sotigalimab/chemotherapy (48.1%, *p* = 0.062) or the triple combination (41.3%, *p* = 0.223). Biomarker analyses suggested treatment-specific correlates: survival after sotigalimab/chemotherapy associated with intratumoral CD4+ T-cell infiltration, circulating differentiated CD4+ T cells, and increased APC numbers [123].

In OPTIMIZE-1, mitazalimab plus mFOLFIRINOX in previously untreated metastatic PDAC produced a confirmed ORR of 40% (23/57; updated confirmed ORR 42.1%), median PFS of 7.7 months, median OS of 14.3 months, and median duration of response of 12.6 months. One-year PFS and OS were 34% and 59%, respectively [118,121]. Objective responders showed intratumoral myeloid and T-cell activation, and mitazalimab-induced expansion of effector CD4+ T cells after the priming dose correlated with improved outcomes. No cytokine release syndrome was reported, and the safety profile was broadly consistent with mFOLFIRINOX, without hepatotoxicity-related treatment discontinuations [118].

Across early-phase studies, chemotherapy–CD40 combinations have been feasible and generally manageable [118,120,122]. Cytokine release syndrome with selicrelumab and sotigalimab was mostly grade 1–2, and liver function test elevations were transient and dose dependent. Grade ≥ adverse events were mainly hematologic, consistent with the chemotherapy backbone. These data justify continued randomized evaluation, but they also show that chemotherapy choice, sequencing, and timing of CD40 agonist administration are likely critical determinants of efficacy and tolerability. The planned randomized phase III trial of mitazalimab plus mFOLFIRINOX will be important to determine whether OPTIMIZE-1 translates into a survival advantage over standard chemotherapy [118,121].

### 4.3. CD40 Agonism Combined with Immune Checkpoint Inhibition

PD-1/PD-L1 and CTLA-4 blockade have limited efficacy in PDAC because baseline priming is weak and myeloid suppression is dominant. CD40 agonism may improve checkpoint responsiveness by licensing APCs, expanding tumor-specific T-cell priming, and shifting macrophages away from suppressive programs [27,125,126]. Preclinical models support durable control and immune memory with CD40-ICI combinations [94,125,126]. In a therapy-resistant pancreatic cancer model, CD40 agonism plus checkpoint inhibition induced complete tumor regression, whereas checkpoint inhibition alone did not [126]. In another murine model, CD40 agonism plus checkpoint blockade cured 63% of tumor-bearing animals and increased tumor-specific T cells in the pancreas [127]. Similar findings in an orthotopic Kras/Trp53-mutant model showed reduced tumor burden, enhanced DC migration, and fewer suppressive TAMs [94].

Clinically, the phase Ib PRINCE trial tested sotigalimab with gemcitabine/nab-paclitaxel, with or without nivolumab, in untreated metastatic PDAC [122]. Treatment was feasible and showed encouraging activity in 24 DLT-evaluable patients, with a cumulative ORR of 58%, median PFS of 11.7 months, median OS of 20.1 months, and 1-year PFS and OS of 44% and 70%, respectively [122].

The randomized phase II PRINCE expansion (n = 105), however, did not show a survival benefit for the CD40-containing arms compared with chemotherapy alone [123]. The 1-year OS endpoint was met only for nivolumab plus chemotherapy (57.7% versus 35% historical rate; *p* = 0.006; n = 34), whereas sotigalimab plus chemotherapy showed a non-significant signal (48.1%; *p* = 0.062; n = 36), and the triple combination did not meet the endpoint (41.3%; *p* = 0.223; n = 35). Survival after sotigalimab/chemotherapy correlated with CD4+ T-cell infiltration, circulating differentiated CD4+ T cells, and APCs; survival after nivolumab/chemotherapy correlated with a less suppressive baseline TME and more activated, antigen-experienced circulating T cells. No subset clearly benefited from the triple combination, possibly because layered immune activation induced exhaustion [123].

CDX-1140, a fully human IgG2 CD40 agonist designed to activate CD40 without Fc-gammaR crosslinking, has also been tested in combinations. A completed phase I trial (NCT03329950) evaluated CDX-1140 alone or with CDX-301 (recombinant human Flt3L), pembrolizumab, or gemcitabine/nab-paclitaxel in advanced malignancies, including PDAC. Preliminary conference data suggested tolerability and immune activation [128,129].

A separate randomized, open-label phase II trial (NCT04536077) of CDX-1140, with or without CDX-301, in PDAC was terminated before completion. Although no peer-reviewed clinical publication is available, preliminary translational data indicate that low tissue Flt3L levels contribute to conventional DC deficits in PDAC and that combined Flt3L and CD40 agonism can restore cDC numbers and function in mouse models and patient samples [130]. However, the same approach also increased regulatory T cells through cDC2 activation, potentially dampening immunity. These findings illustrate both the promise and complexity of DC-centered CD40 combinations.

LVGN7409, a humanized agonistic CD40 antibody, is being evaluated in an active phase I study (NCT04635995) as monotherapy and with anti-PD-1 and/or a CD137 agonist in advanced or metastatic solid tumors, potentially including PDAC. Preliminary monotherapy data from 12 heavily pretreated patients showed no dose-limiting toxicities; among 9 evaluable patients, 44% achieved stable disease [131]. The CD137 (4-1BB) combination is mechanistically relevant because it may amplify T-cell effector function and survival after CD40-licensed priming [132].

A phase Ib/II trial (NCT05419479) assessed sotigalimab, domvanalimab (anti-TIGIT), and zimberelimab (anti-PD-1) in metastatic PDAC. The trial is currently suspended, and no results have been published, again illustrating the operational and biological challenges of multi-agent immunotherapy in PDAC.

Together, these trials show that CD40 agonism does not automatically overcome PDAC resistance to checkpoint blockade. In unselected patients, adding checkpoint inhibition to CD40 agonism and chemotherapy may be insufficient, and excessive immune layering may even promote exhaustion rather than an additive benefit [123]. Future strategies should define the drivers of exhaustion, test mitigation approaches [133], and use biomarkers to select patients most likely to benefit.

### 4.4. Integration with Cancer Vaccines and Neoantigen-Directed Therapies

Vaccine strategies in PDAC, including personalized neoantigen approaches and KRAS-targeted vaccines, aim to generate de novo tumor-specific T cell responses against epitopes less constrained by tolerance [134,135]. CD40 agonism is central to DC maturation and antigen cross-presentation, and IL-12-associated Th1 programming, and may increase the magnitude and functional quality of vaccine-induced responses by enhancing antigen uptake, processing, and presentation. This rationale was confirmed in a preclinical model, in which CD40 and CD80/86 signaling in cDC1s played a critical role in the effective antitumor immunity conferred by a neoantigen-based therapeutic vaccine [136]. In a murine pancreatic cancer model, DC vaccination in combination with a CD40 agonist was necessary to improve survival in an advanced PDAC setting, whereas CD40 agonism alone was ineffective [96].

The phase I REACtiVe-2 trial evaluated MesoPher, a monocyte-derived DC vaccine pulsed with allogeneic tumor lysate, combined with mitazalimab after completion of (m)FOLFIRINOX in metastatic PDAC (n = 16) [124]. Treatment increased activated and vaccine-specific T-cell responses systemically and was associated with increased T-cell infiltration and decreased collagen deposition in post-treatment metastatic biopsies. No objective radiological responses were observed, but 50% of patients had stable disease after three administrations. Because 50% already had progressive disease at baseline, the study underscores that patient selection and disease tempo may be decisive for vaccine-CD40 strategies.

Another vaccination trial (NCT02600949) is testing a personalized neoantigen peptide vaccine alone or with imiquimod, pembrolizumab, and/or sotigalimab in advanced pancreatic and colorectal cancers. Irreversible electroporation (IRE), although not a conventional vaccine, may serve as an in situ antigen-release platform by inducing immunogenic cell death and tumor antigen release [137]. A phase I trial (NCT06205849) is evaluating surgical IRE with intratumoral mitazalimab in locally advanced pancreatic cancer, leveraging local antigen release together with CD40-mediated APC licensing. This approach may inform locoregional immunotherapy strategies for LAPC.

Overall, vaccines and CD40 agonists target complementary bottlenecks in PDAC immunity. Vaccines provide defined antigen targets, whereas CD40 activation licenses APCs and supplies co-stimulation needed for effective T-cell priming. The key unanswered questions are timing, patient selection, disease setting, and whether these combinations can overcome stromal and myeloid barriers sufficiently to generate durable benefit.

### 4.5. Neoadjuvant and Window-of-Opportunity Experience

Window-of-opportunity and neoadjuvant studies are valuable because they allow direct measurement of intratumoral pharmacodynamics, while immune trafficking and lymphatic drainage may be more intact than in widely metastatic disease. Neoadjuvant selicrelumab has demonstrated immune activation and TME remodeling in human PDAC, providing proof of mechanism, although the relationship with long-term outcomes remains unresolved [42]. These settings are well-suited to optimize sequencing and validate tissue-based pharmacodynamic endpoints for later trials.

### 4.6. Biomarkers: Prognostic Context and Predictors of Benefit

Pan-cancer studies have linked higher CD40 expression to improved survival in some settings, but this association is inconsistent and appears limited in PDAC [29,138,139,140,141]. In PDAC transcriptomic analyses, CD40 mRNA is not a robust independent predictor of OS, and CD40 expression in tumor cells has not demonstrated consistent prognostic value in larger patient cohorts [29,142].

In a spatial multi-cohort analysis across nine solid tumors, CD40 expression was present in 68% of pancreatic adenocarcinomas, but tumor-cell CD40 expression was not prognostic for OS [29]. Similarly, a pan-cancer transcriptomic analysis found high CD40 RNA expression in 42% of pancreatic cancers, and an association with improved OS among patients receiving ICIs, but this association was not observed in multivariable analysis, arguing against CD40 as an independent predictive biomarker [142]. Soluble markers may have more prognostic value: high serum soluble CD40 (sCD40) was associated with shorter OS, particularly after neoadjuvant chemotherapy, and improved diagnostic value when combined with CA19-9 [143]. High soluble CD40L (sCD40L) was also associated with poor survival, unresectability, and distant metastasis, and performed better than CA19-9 and CEA as a prognostic marker in one study [103].

Outcome associations in CD40-treated PDAC more consistently reflect immune composition and functional state than baseline CD40 abundance, particularly DC infiltration, macrophage polarization, and treatment-induced immune remodeling [42,121,123]. Neoadjuvant and adjuvant selicrelumab increased circulating CD4+ and CD8+ T cells, elevated inflammatory cytokines, reduced tumor fibrosis, decreased M2-like macrophages, increased mature DCs, and enriched intratumoral T cells [42]. In PRINCE, higher baseline frequencies of circulating DCs, B cells, and experienced Th1 cells were associated with improved survival after sotigalimab plus gemcitabine/nab-paclitaxel; intratumoral Th1, Th2, and IFN-gamma response signatures, as well as specific CD4+ T-cell states, were also associated with survival [123]. In OPTIMIZE-1, baseline tumor-intrinsic fibrosis and ECM-remodeling gene signatures were associated with improved survival after mitazalimab plus mFOLFIRINOX, alongside increased activation and proliferation of circulating T cells and NK cells [121].

These data support prioritizing on-treatment pharmacodynamic biomarkers, APC activation, myeloid polarization, T-cell activation and recruitment, immune infiltration, and stromal remodeling over baseline CD40 abundance alone.

### 4.7. Safety Considerations and Toxicity Management

CD40 agonists can cause systemic immune activation with cytokine-associated symptoms, transient cytopenias, and elevations in liver enzymes; cytokine release syndrome is usually moderate and transient [27,35,42,89,144,145]. Transaminase elevations often occur within approximately 24 h of dosing and may persist for weeks before resolving, supporting close laboratory monitoring and dosing strategies that balance potency with tolerability [35,42,89,144,145]. Safety varies among agents, likely reflecting Fc-gammaR engagement, clustering requirements, and dosing schedules. In PDAC combination studies, including REACtiVe-2, tolerability has generally been manageable, supporting further development while emphasizing the need for regimen optimization [42,118,120,122,123,124].

## 5. Challenges and Future Directions

### 5.1. Barriers to Consistent Clinical Benefit

Despite strong biology, the durable efficacy of CD40 agonism in PDAC remains difficult to achieve. Major barriers include baseline myeloid dominance, limited endogenous priming, dense desmoplasia, and abnormal vasculature, which can continue to restrict effector trafficking and function even when APC activation occurs [4,93]. CD40 agonists also differ in clustering requirements, Fc-gammaR dependence, and exposure profiles, leading to agent-specific pharmacodynamics and safety profiles [114,146]. Outcomes are further shaped by disease setting, tumor burden, immune composition, and treatment sequencing [42,75,118,122,123]. Finally, adaptive counter-regulation, suppression of myeloid re-emergence, regulatory T-cell expansion, compensatory inhibitory pathways, and stromal reconstitution may limit durability and argue for longitudinal tissue-based trial designs [113,127,130].

### 5.2. Biomarkers and Patient Selection: Beyond Baseline CD40 Abundance

Available data do not support baseline CD40 expression as a reliable prognostic or predictive biomarker in PDAC [29,142]. Future stratification should focus on the functional and spatial state of the TME, including DC subset abundance and activation [127,130], macrophage polarization [113], TLS features [147,148], and stromal programs [121]. High-dimensional approaches, including single-cell profiling, multiplex tissue imaging, spatial transcriptomics/proteomics, and longitudinal immune monitoring, can define tumor-immune states permissive to CD40-driven priming and identify early pharmacodynamic correlates of benefit [149,150,151]. Practically, trials should emphasize on-treatment endpoints such as APC activation signatures, myeloid reprogramming, trafficking chemokines, immune infiltration, and stromal remodeling [42,75,121].

### 5.3. Optimization of Dosing, Scheduling, and Delivery

The therapeutic window of CD40 agonists remains a key constraint. Dosing should maximize intratumoral pharmacodynamics while limiting systemic inflammatory toxicity [152]. Step-up dosing, intermittent schedules, and combination-specific dose adjustment should be tested with mechanistic readouts that define exposure–response relationships, including APC activation and cytokine programs [118,145]. Locoregional or intratumoral delivery may improve spatial specificity and reduce systemic exposure [34,153], but in PDAC, this requires feasibility testing and proof that local activation generates systemic antitumor immunity [53,154].

### 5.4. Context-Dependent Tumor-Intrinsic CD40 Signaling

Tumor-intrinsic CD40 signaling remains an unresolved translational issue. Depending on tumor state and microenvironmental cues, NF-kB/MAPK programs may drive apoptosis or pro-survival/proliferative effects [28,109,110]. Because CD40 agonists may engage tumor-cell CD40 in a subset of PDACs [29,142], future work should define how genomic alterations, epigenetic states, and cytokine signals shape tumor-cell CD40 responses [92,155]. This will determine whether tumor-cell CD40 status should inform patient selection or combination design to avoid unintended protumor signaling [32,39].

### 5.5. Next-Generation CD40 Agonists and Rational Combinations

Next-generation formats, including Fc-engineered antibodies [156,157], multivalent ligand–mimetic constructs [158], bispecific or conditional agonists [32,154,159,160], and tumor-localizing delivery systems [53,153,161], aim to improve productive receptor clustering in the relevant immune niche while reducing systemic activation. CD40 agonism is unlikely to be sufficient as monotherapy and should be incorporated into regimens that align antigen supply, APC licensing, and the relief of downstream suppression [42,96,118,130]. Chemotherapy and radiotherapy can induce antigen release and microenvironmental modulation [118,122,162], checkpoint blockade may require demonstrable priming [94,123,126,163], and vaccine/DC/neoantigen strategies can focus T-cell responses [96,124]. These combinations may be most informative in neoadjuvant or minimal residual disease settings, where tumor burden is lower and immune trafficking may be less constrained [42,96].

### 5.6. Standardization of CD40 Measurement and Reporting

CD40 measurement must be standardized to enable comparison across studies. Bulk transcriptomics measures CD40 mRNA in mixed tissue and is sensitive to immune/stromal admixture [142]. Spatial profiling shows multicellular heterogeneity and treatment-induced compartmental shifts that can alter bulk signals without reflecting true cell-intrinsic change [150,164]. Protein-level assays such as IHC, multiplex IF, and quantitative immunofluorescence improve compartment attribution but vary across antibody clones, platforms, scoring methods, sampling, and intratumoral heterogeneity [107,165]. Studies should therefore report assay modality, scored compartment, scoring threshold, specimen type, disease site, and treatment context, and should integrate bulk with spatially resolved approaches when feasible [150,151,164].

## 6. Conclusions

CD40 is a multicompartment immunotherapeutic target in PDAC. It can license APCs, reprogram suppressive myeloid states, and contribute to stromal and vascular remodeling, thereby addressing key mechanisms of immune exclusion. Early studies show clear pharmacodynamic immune engagement, but clinical benefit remains inconsistent, and baseline CD40 expression alone is insufficient for patient selection. Progress will depend on agent-specific optimization of clustering and Fc-gammaR biology, better sequencing with antigen-releasing therapies, on-treatment spatial and pharmacodynamic biomarkers, and safer dosing or tumor-localized delivery strategies.

A particularly promising direction is to combine CD40 agonism with next-generation cancer vaccines, including mRNA-based, KRAS-targeted, neoantigen-directed, and dendritic cell-based platforms. In this setting, CD40 activation may provide the APC licensing and myeloid reprogramming required to convert vaccine-induced antigen recognition into durable T-cell priming within the hostile PDAC microenvironment. Dedicated studies are needed to define timing, partners, biomarkers, and patient selection. With these refinements, CD40-directed therapy remains a biologically compelling strategy to increase the proportion of patients with PDAC who achieve meaningful immune-mediated tumor control.

## Figures and Tables

**Table 1:** Monoclonal CD40 agonists utilized in clinical trials with pancreatic cancer.

Agent	Other Names	Developer	Antibody Class	Fc Engineering
**Sotigalimab**	APX005M	Apexigen/Pyxis Oncology	IgG1	Enhanced FcγRIIB binding
**Mitazalimab**	JNJ-64457107/ADC-1013	Alligator Bioscience	IgG1	FcγR crosslinking-dependent; no specific Fc engineering reported
**Selicrelumab**	RG7876/CP-870893	Pfizer/Genentech/Roche	IgG2	None
**CDX-1140**	-	Celldex Therapeutics	IgG2	None
**ChiLob 7/4**	-	University of Southampton	IgG1	None
**SEA-CD40**	-	Seagen	IgG1	Non-fucosylated Fc; Enhanced FcγRIIIa binding
**LVGN7409**	-	Lyvgen Biopharma	IgG1	Selective FcγRIIB binding

**Table 2:** Completed clinical trials using monoclonal CD40 agonists in pancreatic cancer.

Regimen	Design	Tumor Type	Treatment Scheme	Clinical Outcomes	Immunological Outcomes	Toxicity	Clinical Trial/References
**ChiLob 7/4**	Phase I	CD40^+^ solid tumors and DLBCL, including 2 PDAC (n = 28)	ChiLob7/4 weekly × 4 doses	No objective responses; SD in 52%, with a median duration of 6 months	Immune activation and effector cell trafficking	Well-tolerated; 1 DLT; infusion reactions prevented with single-dose corticosteroid premedication	NCT01561911 [89]
**SEA-CD40**	Phase I	Advanced solid tumors (n = 56) and lymphoma (n = 11), including 3 PDAC (n = 67)	SEA-CD40 monotherapy, 21-day cycle	1 CR and 3 SD in seven lymphoma patients; no PDAC responses reported	Cytokine induction; T and NK cell activation	Acceptable; IHRs in 73%, primarily grade 1–2	NCT02376699 [119]
**CP-870893 (=selicrelumab) + gemcitabine**	Phase I	Chemo-naive advanced PDAC (n = 22)	Gemcitabine weekly × 3 weeks + CP-870893 on day 3 of each 28-day cycle	ORR 19%; mPFS 5.2 months; mOS 8.4 months; 1-year OS 28.6%	Inflammatory cytokine increase; increased costimulatory molecules on B cells; Transient depletion of B cells	Well-tolerated; 1 DLT; grade 1–2 CRS most common	NCT00711191 [120]
**Selicrelumab ± Gem/Nab**	Phase I	Resectable PDAC (n = 16)	(1) neoadjuvant selicrelumab followed by surgery; (2) neoadjuvant Gem/Nab + selicrelumab followed by surgery; Adjuvant Gem/Nab + selicrelumab, up to 4 28-day cycles, in both arms	Combined: mOS 23.4 months; mDFS 13.9 months; (1) mOS 23.4 months; mDFS 9.8 months; 1-year DFS 49.9%; 1-year OS 81.8%; (2) mOS and mDFS not reached; 1-year DFS 75.0%; 1-year OS 100%	T cell enrichment in 82% of tumors; increased active and proliferative T cells; Reduced tumor fibrosis; decreased M2-like macrophages; increased mature intratumoral DCs; increased inflammatory cytokines	Acceptable; 3 SAEs in 2 patients; grade 3–4 AEs in 6 patients	NCT02588443 [42]
**Mitazalimab + mFOLFIRNOX** **(OPTIMIZE-1)**	Phase Ib/II	Chemo-naive mPDAC (n = 70)	Mitazalimab on day 1 (priming dose) and day 10, and mFOLFIRINOX on day 8; subsequent cycles: mFOLFIRINOX on day 1, mitazalimab on day 3	ORR 40%; mPFS 7.7 months; mOS 14.3 months; 1-year PFS 34%; 1-year OS 59%	Activated myeloid, B cell, and T cell frequencies correlated with better outcomes; Intratumoral myeloid and T cell activation in objective responders	Manageable; 1 DLT; SAEs in 41%, not related to mitazalimab; Most common grade ≥3 AEs: neutropenia 26%, hypokalaemia 16%, anaemia and thrombocytopenia 11%	NCT04888312 [118,121]
**Sotigalimab (APX005M) + Gem/Nab +/− nivolumab** **(PRINCE)**	Phase Ib	First line mPDAC (n = 30)	(1) Nivolumab + Gem/Nab; (2) Sotigalimab + Gem/Nab; (3) Sotigalimab + nivolumab + Gem/Nab; Nivolumab on day 1 and 15; Sotigalimab on day 3 (2 days after chemotherapy), or day 10 if chemotherapy on day 8	ORR 58%; mPFS 11.7 months; mOS 20.1 months	B cell shift to plasmablasts; increased CD141^-^ myeloid DCs and pDC frequency; increased activated CD8^+^ and CD4^+^ T cells; decreased *KRAS* VAF in 86%	Tolerable; 2 DLTs; 47% treatment-related SAEs but unrelated to either sotigalimab or nivolumab; 93% grade 3–4 treatment-related AEs (mostly hematologic, transient); 2 Gem/Nab-related deaths; 1 death from an unknown cause 4 months after last study intervention	NCT03214250 [122]
**Sotigalimab (APX005M) + Gem/Nab +/− nivolumab** **(PRINCE)**	Phase II	First line mPDAC (n = 105)	Same 3 arms as phase Ib	(1) ORR 50%; mPFS 6.4 months; mOS 16.7 months; 1-year OS 57.7%; (2) ORR 33%; mPFS 7.3 months; mOS 11.4 months; 1-year OS 48.1%; (3) ORR 31%; mPFS 6.7 months; mOS 10.1 months; 1-year OS 41.3%	Not reported in this phase	98% with ≥1 treatment-related AEs; Most common grade 3–4 treatment related AEs were hematologic and generally transient; 2 treatment-related deaths	NCT03214250 [123]
**Mitazalimab + autologous DC vaccine** **(REACtiVe-2)**	Phase I	mPDAC (n = 16)	25 × 10^6^ DCs (1/3 i.d. and 2/3 i.v.) co-administered with mitazalimab, bi-weekly for max. 5 administrations	No objective responses; SD in 50% after 3 administrations; In patients with non-PD at baseline: mPFS 2.76 months; mOS 12.1 months; 1-year PFS 13%; 1-year OS 50%	Increased vaccine-specific T cell responses; Increased intratumoral T cells; Decreased collagen deposition	Safe; well-tolerated; 1 transient DLT (grade 3 fever)	NCT05650918 [124]

AE, adverse event; CD, cluster of differentiation; CR, complete response; CRS, cytokine release syndrome; DC, dendritic cell; DFS, disease-free survival; DLBCL, diffuse large B cell lymphoma; DLT, dose-limiting toxicity; Gem/Nab, gemcitabine plus nab-paclitaxel; i.d., intradermal; IHR, infusion/hypersensitivity reaction; i.v., intravenous; mDFS, median disease-free survival; mFOLFIRINOX, modified FOLFIRINOX; mOS, median overall survival; mPDAC, metastatic pancreatic ductal adenocarcinoma; mPFS, median progression-free survival; NK, natural killer; ORR, objective response rate; OS, overall survival; PD, progressive disease; PDAC, pancreatic ductal adenocarcinoma; pDC, plasmacytoid dendritic cell; PFS, progression-free survival; SAE, serious adverse event; SD, stable disease; VAF, variant allele frequency.

## Data Availability

No new data were created or analyzed in this study.
